# Impact of moderate exercise on fatty acid oxidation in pancreatic β-cells and skeletal muscle

**DOI:** 10.1007/s40618-021-01551-2

**Published:** 2021-04-12

**Authors:** A. Langlois, A. Forterre, M. Pinget, K. Bouzakri

**Affiliations:** Centre Européen D’étude du Diabète, Unité Mixte de Recherche de L’Université de Strasbourg « Diabète et Thérapeutique », Strasbourg, France

**Keywords:** FA oxidation, Moderate exercise, Pancreatic beta cell, Skeletal muscle

## Abstract

Fatty acids (FA) play a crucial role in glycaemia regulation in healthy and metabolic disorders conditions through various mechanisms. FA oxidation is one of the processes involved in lipid metabolism and can be modulated by exercise. Nowadays, physical activity is known to be an effective strategy for the prevention and treatment of Type 2 Diabetes. Moreover, its intensity, its duration, the sex-gender, the prandial state, exerkines… are as many parameters that can influence glycaemic control. However, the widely debated question is to determine the best type of exercise for patients with metabolic disorders. In this review, we will discuss the impact of exercise intensity, especially moderate activity, on glycaemic control by focussing on FA oxidation in pancreatic β-cells and skeletal muscle. Finally, thanks to all the recent data, we will determine whether moderate physical activity is a good therapeutic strategy and if FA oxidation represents a target of interest to treat diabetic, obese and insulin-resistant patients.

## Introduction

Type 2 Diabetes (T2D)-associated lipotoxicity, due to an increase of circulating fatty acids (FAs), induce skeletal muscles insulin resistance and pancreatic β-cell dysfunction [1–4] However, it is well documented that FAs are essential for several cellular functions (i.e. vesicle exocytosis) and particularly have critical roles on the regulation of glycaemia homeostasis by acting on pancreatic β-cells and skeletal muscle [5–9].

FAs are metabolized to provide regulatory metabolic coupling factors (R-MCFs) and effectory MCFs (E-MCFs). The R-MCFs, like citrate, NADH/NAD^+^, malonyl-CoA, GTP, long-chain Acyl-CoA compounds [FA-CoA], glutamate and adenine nucleotides, promote the synthesis of E-MCFs which are ATP, cAMP, monoacylglycerol, NADPH, ROS, inositol 1,4,5-trisphosphate and short-chain Acyl-CoA compounds. E-MCFs are known to have a direct impact on insulin secretion in pancreatic β-cells [7, 10] and on glucose uptake in skeletal muscle [11]. These, physiological effects of FAs are mediated by three interdependent processes, the FA oxidation, the Triglyceride (TG)/FA cycle and the activation of Gq-coupled FA receptors [6].

Furthermore, FAs represent an important source of energy during exercise [12]. More precisely, the nature of the energy source varies in function of the exercise intensity. At a moderate intensity (VO_2_max < 40%), lipids are predominantly the fuel supplier, whereas carbohydrates are preferentially used in a more intensive physical practice [13]. Nowadays, physical activity is known to be an effective strategy for the prevention and treatment of Type 2 Diabetes [14]. However, the remaining question is to determine what type of exercise is recommended in case of metabolic disorders (Obesity, T2D).

Thus, in this review, we will discuss the impact of exercise intensity, with a focus on moderate activity, on FA oxidation in pancreatic β-cells and skeletal muscle.

## Biochemistry of FA oxidation

In mammals, FA β-oxidation occurs in two different cell compartments: in mitochondria with the production of ATP and in peroxisomes, in which no ATP is produced [15, 16].

### Mitochondrial β-oxidation

**Fig. 1:**
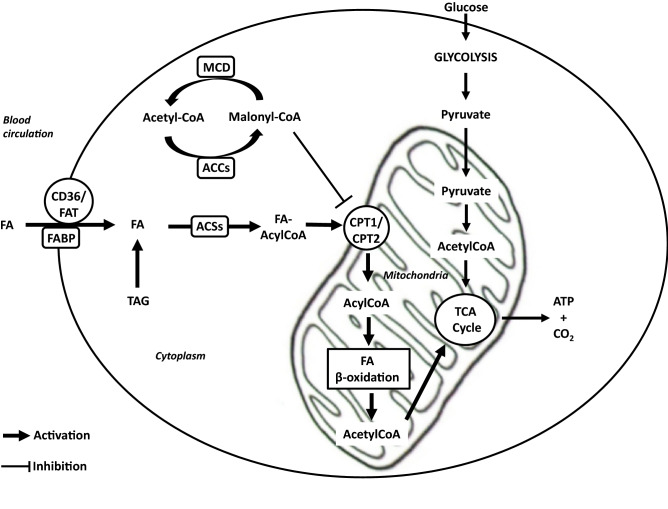
Mechanisms of Fatty acids and glucose mitochondrial β-oxidation. ATP and CO_2_ are produced by cell from glucose and FA pathways. Focussing on lipid metabolism, FAs enter the cell via specific transporters (CD36/FAT, FABP) or are provided from TAG present in the cytosol. Before entering inside mitochondria, FAs are activated in FA-Acyl CoA form by ACS enzyme. After that, CPT1/2 convert FA-Acyl CoA in Acyl-CoA and β-oxidation is also performed to produce Acetyl-CoA. Finally, this latter is used by the TCA cycle to produce ATP and CO_2_ necessary for cell physiology. Malonyl-CoA is a key regulator of this mechanism inhibiting CPT1 and consequently the transfer of FA-AcylCoA into the mitochondria. Malonyl-CoA is produced by ACC and catabolized by MCD. *ATP* Adenosine Triphosphate, *CO*_*2*_ Dioxide carbon, *FA* Fatty acid, *CD36* Cluster of differentiation 36, *FAT* Fatty acid transporter protein, *FABP* Fatty acid binding protein, *FA-Acyl CoA* Fatty acid-Acyl Coenzyme A, *TAG* triacylglycerol, *ACS* Acyl-CoA synthetases, *CPT1/2* Carnitine palmitoyl transferase 1 and 2, *TCA* Tricarboxylic acid, *Malonyl-CoA* Malonyl-Coenzyme A, *ACC* Acetyl-Coenzyme A carboxylase, *MCD* Malonyl coenzyme A decarboxylase

Mitochondrial β-oxidation (Fig. 1) contains several steps which are all regulated by several mechanisms. First, FAs are either provided by nutrients, lipid droplets or as a result of the endogenous triglycerides lipolysis [6, 8]. Particularly, FAs used during exercise can originate from the circulation, packed in triacylglycerol-rich particles provided by the liver or as non-esterified FAs (NEFAs) from adipose tissue lipolysis. However, another source of FA used during exercise is the intramyocellular lipid (IMCL) stored in the skeletal muscle in triacylglycerol-rich lipid droplets [12, 17]. Then, FAs enter the cell through specific transporters, such as the tissue-specific fatty acid transporter protein CD36/FAT and fatty acid binding protein (FABP), which are located at the cell membrane [8, 18, 19]. However, prior to entering the mitochondrial matrix to undergo oxidation, FAs are rapidly activated into their corresponding CoA esters form (FA-AcylCoenzyme A, FA-AcylCoA) by Acyl-CoA synthetases (ACSs) identified at the plasma membrane, in mitochondria and in lipid droplets [7, 20, 21]. Interestingly, the evolution into the β-oxidation pathway is dependent of the isoform of ACSL (long-chain Acyl-CoA synthetases). In particular, ACSL1 is involved in skeletal muscle FA β-oxidation [20] while ACSL3 and ACSL4 are found in pancreatic β-cells [22]. Then, FA-AcylCoAs are converted to FA carnitines by carnitine palmitoyl transferase 1 (CPT1) and are transported from the cytosol across the outer mitochondrial membrane. The exact role of this regulator is to reduce the Long Chain Fatty Acid (LCFA) oxidation and/or its reesterification into triglycerides. Moreover, it has been shown in nonlipogenic tissues, that Malonyl-CoA controls the intracellular energy balance by inhibiting CPT1. Malonyl-CoA is produced by AMPK substrate Acetyl-CoA carboxylase (ACC) and catabolized by Malonyl coenzyme A (CoA) decarboxylase (MCD) [23]. Thus, AMPK play a crucial role in FAs β-oxidation modulating the concentration of Malonyl-CoA by the phosphorylation and the inhibition of ACC. In human, 2 ACCs isoforms exist, ACC1, cytosolic and ACC2, which is anchored to the outer mitochondrial membrane and controls fatty acid β-oxidation [24]. Abu-Elheiga et al. showed a significant decrease of Malonyl-CoA levels, an elevation of lipid oxidation and a reduction of lipid storage in a mice model lacking the ACC2 [25]. Then, at the inner mitochondrial membrane, CPT2 converts fatty acyl carnitine back to fatty acyl-CoA, which enters b-oxidation, and to free carnitine, which returns to the inner mitochondrial space or cytosol [8].

In the mitochondria, FA-AcylCoA is converted into acetyl coenzyme A (acetyl CoA) by the β-oxidation chemical reaction which is repeated 4 times. After each β-oxidation, two-carbon fragments are removed in the form of acetyl CoA [21, 26]. The latter is then oxidized within the tricarboxylic acid (TCA) cycle and ATP is then generated and represents an essential energetic source for several key cellular functions [20]. This pathway is particularly active in liver, heart and skeletal muscle [21]. Each electron removed from these fuel molecules during the two hydrogenation steps are transferred to the oxidized forms of nicotinamide adenine dinucleotide (NAD +) and flavin adenine dinucleotide (FAD2 +). It results respectively NADH and FADH2 which are used by the mitochondrial electron transport chain for generating energy through their oxidative phosphorylation [21, 27].

### Peroxisomal oxidation

In mammals, peroxisomes are essential organelles for the α-oxidation of branched-chain fatty acids and for β-oxidation of long-chain FA (LCFA) [28, 29]. They are the exclusive organelles for the oxidation of very-long-chain fatty acids (VLCFA; > 22 carbons) [30]. Interestingly, peroxisomal and mitochondrial FA β-oxidation are different. In mitochondria, FADH2 produced during the first dehydrogenation step is reoxidized for energy output whereas in peroxisomes, it reacts with O2 to produce H2O2. By the end to reduce the excess of H2O2, the latter is degraded by peroxisomal catalase into H2O and O2 thus decreasing cells oxidative stress [29].

## Impact of FA β-oxidation alteration in pancreatic β cells and skeletal muscle in metabolic diseases

### Impact of FA β-oxidation on beta-cell dysfunction?

Since insulin secretion process requires a significant amount of energy, the well-described canonical signaling pathway [7, 15–18] is also amplified by FA oxidation in physiological conditions [6, 19].

Three fuel-driven metabolic cycles generate metabolic coupling factors in the β-cell which stimulate insulin secretion: the Krebs cycle, the Pyruvate cycle and the Glycerolipid (GL)/FA cycle [7]. The GL/FA cycle and mitochondrial FA β-oxidation are also closely linked to amplify insulin secretion in β-cells. Actually, GL/FA cycle is proposed as a pathway of insulin secretion amplification through the production of lipolytic metabolites by lipogenesis and lipolysis. Hence, that’s going to fuel β-oxidation process and the generation of E-MCFs which activate late effector components of the insulin granules exocytosis machinery like K^+^_ATP_ channel/SUR1/ Ca^2+^, SNAREs, Munc13-1 and cytoskeletal proteins [7, 20–22].

It has been described that the slightest disturbance of FA oxidation balance can have a positive or negative influence on insulin secretion. Indeed, it was found in obese an overexpression of CD36/FAT leading to decrease insulin secretion, impairing exocytosis and reducing granule docking [23]. Furthermore, dysregulation in the lysine acetylation and deacetylation balance of many respiratory chain proteins results in mitochondrial dysfunction and impair insulin secretion [24–27]. Interestingly, overnutrition (major risk factor of type 2 diabetes), oxidative stress or inhibition of SIRT3 (Class III histone deacetylases protein) lead to lysine hyperacetylation, mitochondrial dysfunction [25] and to impair the insulin secretion process [28]. Indeed, it has been found in INS-1E SIRT3 KO beta cell line, a hyperacetylation of the inhibitory factor 1 (IF1), a major regulator of the ATP synthase [29], thus resulting in decreasing the ATP supply, and consequently a defect of insulin secretion [28]. While, during fasting, SIRT3 expression is increased, which promotes FA β-oxidation and insulin secretion [24]. In addition, elevated glucose inhibits CPT1 and β-cell FA oxidation decreasing insulin secretion [30, 31]. All of these data showed that FA oxidation plays a crucial role in blood glucose homeostasis and its alteration contributes to impair the secretion of insulin in metabolic diseases. Indeed, dysregulation of lipolysis (provider of FA for β-oxidation) occurring with over nutrition or T2D may contribute to lipid droplets (LD) accumulation in pancreatic islets resulting in β-cell dysfunction [22]. However, it has been recently demonstrated that, despite FA β-oxidation is suppressed by Sirtuins deacetylation of FoxO1, the glucose-stimulated insulin secretion (GSIS) is sustained in Diabetes [9, 32]. Thus, FA β-oxidation alteration does not seem to be the direct cause of β-cell dysfunction in metabolic disorders. However, since prolonged exposure to high FA amount has negative actions including reduced glucose metabolism, decreased insulin release and a pro-apoptotic effect on β-cells, lipid metabolism has a crucial role on insulin secretion impairment through different pathways. Indeed, FA also regulate β-cell function through the activation of cell surface G-coupled FA receptors (GPCRs) [6, 33–36]. For example, GPR40/FFAR1 and GPR43/FFAR2 [37] Gq subunit activation potentiates GSIS [33, 38–40]. Unlikely, GPR41/FFAR3 activation inhibits GSIS [33, 41].

### Impact of FA β-oxidation on glucose homeostasis in skeletal muscle

Lipid metabolism plays a critical role in regulating glucose homeostasis in skeletal muscle. Indeed, it was widely shown that elevated plasma FA significantly correlates with reduced insulin-stimulated glucose disposal in skeletal muscle in a dose-dependent manner [42–45]. Contrariwise, a plasmatic FA decrease in insulin resistant and/or T2D patients is associated with an increase in insulin sensitivity in the skeletal muscle [46, 47]. More specifically, several studies mentioned that mitochondrial β-oxidation is an important regulator of the glucose homeostasis in the skeletal muscle with the demonstration of the existence of a straight relationship between FA oxidation and insulin resistance. Indeed, it was demonstrated for years that oxidative phosphorylation and lipid oxidation are both decreased in T2D mellitus which indicates that mitochondrial dysfunction can lead to insulin resistance [48, 49]. Moreover, it was shown in in vivo and ex vivo studies, performed on skeletal muscle of insulin-resistant and T2D subjects that mitochondrial function is impaired [50–53].

For years, whether the FA oxidation alteration (mitochondrial dysfunction) is the trigger or not of insulin signaling impairment inducing insulin resistance onset was the subject of extensive discussions [50, 52, 54, 55]. Recent studies seem to indicate that mitochondrial function impairment is more likely the cause of metabolic disorders. Indeed, Daniele et al., showed that the improvement in insulin sensitivity was closely correlated with the decrease of plasmatic FA, the increases of mitochondrial ATP synthesis > 50% and of insulin-mediated glucose disposal in obese normal glucose tolerant and T2D subjects [50]. Moreover, Toledo et al. showed that skeletal muscle mitochondria are significantly resilient to nutrient overload. More precisely, the authors demonstrated from a cohort of healthy volunteers who underwent 2 month high-fat overfeeding that lower skeletal muscle mitochondrial oxidative capacity observed in obese patients is likely to be caused by reasons other than nutrient overload [54].

These new data indicate that the alteration of FA oxidation is the cause of metabolic disorders-related insulin resistance in skeletal muscle unlike what is described for insulin secretion by pancreatic β-cells. Thus, it would suggest that additional factors may be involved in β-cell dysfunction and in metabolic diseases onset such as myokines secreted by skeletal muscles.

## Impact of myokines on pancreatic β-cell and FA metabolism

Skeletal muscle has been found to secrete several hormones, called myokines, able to impact β cell function and FA metabolism. Indeed, the notion that a muscle-pancreas crosstalk exists has been widely accepted [56–58]. This communication between skeletal muscle and β-cells involves a different panel of myokines expressed and released by myotubes. Moreover, each panel exerts differential effects on β-cells that is modulated by insulin resistance. Thus, it could contribute as well as to normal β-cell functional mass in healthy subjects, as its decrease in type 2 diabetes [59]. Indeed, Bouzakri et al*.* [59] detected increased GSIS in primary human and rat beta cells incubated with conditioned media from human myotubes which contain several factors (myokines, metabolites, exosomes…) [58]. Interestingly, it has been shown that human skeletal muscle cells secrete different myokines depending on their insulin sensitivity and that have a bimodal impact on β-cell insulin secretion, proliferation and survival [59, 60]. For example, Rutti S et al.found that CX3CL1 (fractalkine) is a myokine which protects β-cells from the negative impact of TNFα [60]. Furthermore, Chaweewannakorn C et al. showed that fractalkine, triggered by muscle contractile activity, is required for achieving proper GLUT4 translocation and glucose uptake in skeletal muscle [61]. Thus, myokines can impact β-cell function and glucose homeostasis in skeletal muscle.

In recent years, it was proposed that serum FA levels would have an impact on myokines secretion and consequently, on β-cell function and glucose homeostasis. Indeed, Xu X et al., [62] demonstrated that FSTL-1, an adipo-myokine [63], plays a role in glucose and lipid metabolisms whose circulating concentration is decreased when serum FA level is high. In addition, their results indicate that increase in FSTL-1 secretion is associated with insulin resistance [62]. Similarly, Ordelheide AM et al. [64] identified another FA-induced myokine, named angiopoietin-like protein 4 (ANGPTL4), which has lipolytic properties in humans in vivo [64, 65]. Thus, all these data indicate the existence of an interaction between FA metabolism, myokines and glycaemic control.

At last, physical activity is well known to prevent metabolic diseases [66] and to increase myokine secretion [67]. Indeed, in 2004, the American Diabetes Association recommended for T2D patients a weekly 150 min of moderate to vigorous intensity exercise [128] since physical inactivity is an important risk factor linked to diabetes onset [129]. Moreover, exercise status, its intensity and its duration can positively or negatively modulate the lipid oxidation process [68] and consequently could impact glycaemic control and metabolic diseases onset. Nowadays, the question raised is whether moderate intensity is sufficient to prevent or to cure metabolic disorders. Thus, we will discuss afterwhile whether moderate physical activity intensity is enough to improve metabolic control with a focus on its effect on FA oxidation in β-cell and skeletal muscle.

## Effect of moderate exercise on β-cell function and glucose homeostasis in skeletal muscle: focus on FA oxidation

### Physiology of moderate exercise impact on FA oxidation

Almost a century ago it was already observed that FA oxidation increased 5–tenfold above resting levels during mild to moderate exercise [69, 70]. Conversely, FA oxidation progressively decreased as the intensity of the exercise increases [70]. Afterward, maximal FA oxidation (MFO) occurs during submaximal exercise intensities ranging from 45 to 65% VO_2_max while at higher exercise intensity exceeding MFO, FA oxidation decreases [68]. This process is described as the crossover concept [71, 72]. Interestingly, it was demonstrated that the MFO kinetic is also dependent on the training status, ranging from 23 to 89% VO_2_max [73]. Indeed, it was largely mentioned that trained subjects possess a greater ability to oxidize fat at higher exercise intensities [74, 75]. Additionally, recently it was mentioned that a prolonged exercise for several hours from a low to moderate intensity enhances FA utilization at the expense of glucose as fuel. This is associated with a decrease in glucose availability [76]. Thus, a correlation between respiratory capacity and MFO exists. In this way, Purdom T et al. have suggested that increased cellular respiration capacity with training could enhance FA oxidation at higher exercise intensities [68]. This is emphasized by Cui X et al. who showed that endurance exercise training can promote mitochondrial biogenesis in skeletal muscle and enhance muscle oxidative capacity [49].

To sum up, FA oxidation level is modulated as regards of the physical activity intensity.

### Impact of moderate exercise on β-cell function and FA oxidation

For years, studies reported that exercise impacts insulin secretion and glucose homeostasis regulation [77–80]. Moreover, exercises interventions have been shown to prevent pancreatic β-cell failure in T2D patients [77, 81]. However, the effect of physical activity on β-cell function is dependent on its intensity. Currently, this is well established that moderate-intensity exercise training improves insulin secretion and its action [82]. On one hand, short-term training improves β cell function and efficiently reduces ectopic fat within the pancreas in prediabetic or T2D patients [83]. In addition, studies performed on T2D suggest that long duration-higher intensive exercise are less beneficial than moderate duration and intensive exercises [82]. On the other hand, it is advised that a linear relationship between exercise dose and β-cell function exists in a healthy population [84]. High-intensity training has negative effects on the pancreatic islet in comparison to a moderate intensity one with a reduction in β-cells percentage per pancreatic islet [85]. Finally, this is suggesting that the impact of exercise on β-cell function is dependent on the physiological state of the individuals. Indeed, Dela F et al. observed a significant increase in β-cell responses in hyperglycaemic condition in trained T2D patients, only in the situation in which the remaining secretory capacity is moderate, unlike if the latter is low [86]. Consequently, by considering all these data, it appears essential that exercise intensity have to be adapted in function of the patient health status and that moderate physical practice suits more to improve β-cell function in diabetic patients.

Remarkably, as the principal fuel supplier during moderate exercise intensity are lipids [13], its metabolism and especially FA oxidation would be involved in improving β-cell function in T2D individuals.

For quite a long time, it has been proposed that exercise did not cause any change in the islets lipid metabolism such as FA oxidation. Nevertheless, it significantly reduced pancreatic islets exposure to circulating lipids known to be toxic at high level [72]. Indeed, the authors showed that moderate training on female healthy rats decreased the plasmatic amount of glycerol, FA, and triglycerides. This resulted in the reduction of lipolysis from adipose tissue without alterations of FA oxidation and expressions of key lipid metabolism transcription factors and enzymes (FFAR1, CD36, CPT-1, MCD…) in pancreatic islets. In parallel, it was demonstrated that the training did not alter the glucose-induced insulin secretion or the FA amplification process. More recently and in accordance with these observations, Delghingaro-Augusto V et al. have emphasized that the alteration of β-cell FA metabolism in an in vivo study using Zucker Diabetic Fatty rat, is responsible for the β-cells function failure. Then, the authors showed that a defect in FA oxidation, GL/FA cycling, and β-cell gene expression are still observed in ZDF pancreatic islets despite a voluntary running exercise for 6 weeks as previously observed in inactive ZDF rats. However, islet insulin mRNA and insulin stores were preserved upon exercise in ZDF rats [73].

Thus, these data suggest a potential mechanism by which exercise could prevent the loss of β cells function that leads to T2D [72]. Indeed, Ellingsgaard et al. [87] showed that β-cell function was improved by the increase of the myokine IL-6 following exercise which acts on intestinal L cells and pancreatic α-cells to stimulate GLP-1 release, inducing insulin secretion afterwhile. Additionally, it was shown that exercise protect β-cell viability through IL6 direct action on β-cells [88–91].

**Fig. 2:**
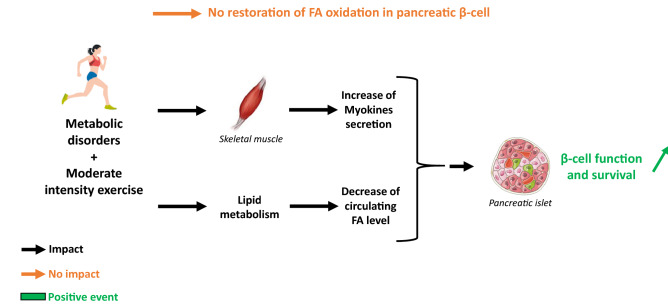
Impact of moderate exercise on pancreatic β-cell in patients with metabolic disorders: focus on FA oxidation, β-cell function and survival. In β-cells, FA oxidation is decreased in people with metabolic disorders. Moreover, physical activity can improve their metabolic control. However, moderate exercise does not allow to restore physiological FA oxidation despite an improvement of β-cell function and survival in diabetic patients. Thus, others mechanisms are involved, such as skeletal muscle-secreted myokines and lipid metabolism, which are described to have exercise-induced beneficial effects. *FA* Fatty acid

In conclusion, all these results indicate that exercise does impact β-cell function through plasmatic content modification (FA, myokines levels…) and not by altering FA oxidation (Fig. 2).

### Impact of moderate exercise on glucose homeostasis in skeletal muscle and FA oxidation

Nowadays physical activity is strongly considered as an effective strategy in both preventing and treating T2D [92, 93]. Indeed, exercise has been largely demonstrated to improve the peripheral insulin sensitivity in T2D patients and to have a beneficial effect on insulin resistance [14, 77–79]. In addition, exercise increases the expression of GLUT 4 in skeletal muscles and thus enhances insulin- and muscle contraction-stimulated glucose uptake into the muscle cells [76, 94]. Furthermore, it was recently reviewed that intramyocellular lipids (IMCL) content in skeletal muscle is increased and fat oxidative capacity decreased in obese people and T2D [12]. This increase of IMCL is also observed in training athletes and this is qualified as the “athlete’s paradox” [95]. However, these use largely more IMCL as an energy source during exercise as compared to obese or T2D who utilized preferentially FA from the blood circulation [96, 97]. Interestingly, this impairment of mitochondrial function observed in insulin-resistant skeletal muscle and T2D is reversible thanks to physical activity. Indeed, it was largely demonstrated that endurance exercise improved mitochondrial respiratory capacity and FA oxidation in T2D and obese people [98, 99]. In addition, Lipid droplet–mitochondria tethering is increased in the muscle of training athletes upon a single bout of exercise and also upon endurance training in obese participants. Moreover, low-volume high-intensity interval training can rapidly improve glucose control and it was suggested that it could be partly mediated by improving the skeletal muscle mitochondrial function [100, 101]. To complete this data, it was recently demonstrated that mitochondrial dynamics and quality control in skeletal muscle seems to be linked to oxidative capacity in humans and this could be involved in the maintenance of insulin sensitivity [102]. In particular, the mitochondrial dynamic proteins OPA1 (responsible for mitochondrial membranes fusion) and FIS1 (involved in mitochondrial fission) are positively correlated with peripheral insulin sensitivity. These expressions are increased in endurance-trained athletes while these are downregulated in T2D individuals [102]. Thus, mitochondrial dysfunction is involved in metabolic disorders and can be reversed by exercise.

Therefore, as moderate exercise increases mitochondrial FA oxidation it has been suggested that it could be beneficial for the glucose metabolism regulation in diabetic patients. However, Chavanelle V et al. [14] have recently demonstrated in a diabetic mice db/db model exposed to 2 kind of intensity exercises, that high-intensity physical activity presents a lowered fasting glycaemia and HbA1c level as compared to resting and moderate activity conditions. Consequently, it seems that moderate exercise has no positive effect on glycaemic control in diabetic conditions despite an enhancement of FA oxidation as previously described. Moreover, no matter which exercise intensity is performed, no effect was observed in the mitochondrial function markers assessed (TFAM, PPAR-α…). So, these data indicate that there are no functional adaptations of mitochondria following chronic exercise and others mechanisms should be involved to explain the beneficial effect of high intensity training on glycaemic control. Furthermore, a significant increase of muscle Glut4 content and higher insulin-stimulated Akt phosphorylation ratios was only obtained in diabetic mice under high intensity physical activity [14]. Thus, it is suggested that in T2D, stimulating insulin signalling and Glut4 content in muscle is an important strategy to improve glycaemic control and that high intensity exercise would be the solution rather than moderate activity. Nevertheless, according to authors [14] it seems important to confirm such data in a more physiological relevant animal model of obesity and T2D, as in db/db mice in which the leptin receptor gene is mutated [103].

**Fig. 3:**
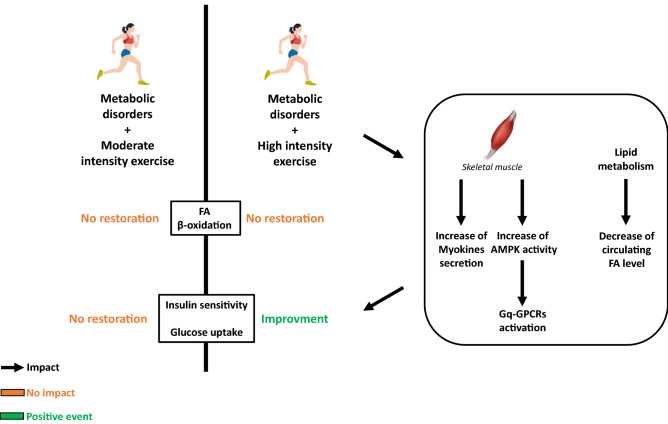
Impact of moderate exercise in skeletal muscle of patients with metabolic disorders: focus on FA oxidation, insulin sensitivity and glucose uptake. In skeletal muscle, FA oxidation and glycaemic control are altered in people with metabolic disorders. Exercise improves peripheral insulin sensitivity in T2D patients and has a beneficial effect on insulin resistance. However, only high intensive exercise improves metabolic control in skeletal muscle. This beneficial effect is not related to its impact on FA oxidation but dependent to skeletal muscle-secreted myokines, plasmatic FA level and to the activation of specific Gq-GPCRs. *FA* Fatty acid, *Gq-GPCRs* subunit Gq-G-coupled FA receptors, *AMPK* Adenosin monophosphate-activated protein kinase

To conclude, by considering all these data, the beneficial effect of exercise (only at high intensity) on metabolic control in skeletal muscle is not related to its impact on FA oxidation but could rather be dependent to the plasma content (Myokines, FA levels…) and/or to the activation of specific GPCR, as previously described for β-cells. Indeed, action of myokines expressed and secreted during exercise has been shown to improve insulin sensitivity in T2D patients [59, 104]. Furthermore, it is well established that skeletal muscle GPCRs are involved in glucose uptake and whole-body glucose homeostasis [105–107]. Interestingly, Bone DBJ et al. found in skeletal muscle that Gq-GPCRs activation promotes glucose uptake and improves glucose homeostasis in obese, glucose-intolerant mice through the activity of AMPK (which increases with exercise). Similarly, Gq-GPCR activation stimulates glucose uptake in primary human skeletal muscle cells [105] **(**Fig. 3).

## Conclusion

In this review, we gathered recent data which permit to determine the real impact of moderate exercise on pancreatic β-cells function and glucose homeostasis in skeletal muscle by focusing on FA oxidation. Recent studies seem to demonstrate that FA oxidation alteration may not be the direct cause of β-cell dysfunction in metabolic disorders. Conversely, alteration of FA oxidation is the cause of metabolic disorders-related insulin resistance in skeletal muscle. Then, in comparison to high intensity, moderate exercise increases FA oxidation in skeletal muscle. Despite this increase, a better impact on insulin sensitivity is observed with a more intensive activity. Moreover, it appeared that exercise positively impacts glycaemic control (β-cell function and glucose homeostasis in skeletal muscle) at high intensity by altering FA oxidation but through plasmatic content modification (Myokines, FA levels…) and/or Gq-GPCRs activation. Nevertheless, the intensity is not the only parameter to influence physical activity efficiency in metabolic control. Duration, sex-gender, prandial state, exercise metabolites called “exerkines” (hormones, myokines…) can also impact glycaemic regulation. At last, further studies are necessary to understand precisely the mechanisms involved, as these parameters represent a great interest in the development of future strategies for the treatment and patients' medical care for metabolic diseases.
